# Characterization and utilization of the flexor digitorum brevis for assessing skeletal muscle function

**DOI:** 10.1186/s13395-018-0160-3

**Published:** 2018-04-17

**Authors:** Michael D. Tarpey, Adam J. Amorese, Nicholas P. Balestrieri, Terence E. Ryan, Cameron A. Schmidt, Joseph M. McClung, Espen E. Spangenburg

**Affiliations:** 1 Department of Physiology, Brody School of Medicine, East Carolina University, Greenville, NC 27834 USA; 2 East Carolina Diabetes and Obesity Institute, East Carolina University, Greenville, NC 27834 USA

**Keywords:** cDNA electroporation, Mitochondrial respiration, Muscle stimulation, Skeletal muscle function

## Abstract

**Background:**

The ability to assess skeletal muscle function and delineate regulatory mechanisms is essential to uncovering therapeutic approaches that preserve functional independence in a disease state. Skeletal muscle provides distinct experimental challenges due to inherent differences across muscle groups, including fiber type and size that may limit experimental approaches. The flexor digitorum brevis (FDB) possesses numerous properties that offer the investigator a high degree of experimental flexibility to address specific hypotheses. To date, surprisingly few studies have taken advantage of the FDB to investigate mechanisms regulating skeletal muscle function. The purpose of this study was to characterize and experimentally demonstrate the value of the FDB muscle for scientific investigations.

**Methods:**

First, we characterized the FDB phenotype and provide reference comparisons to skeletal muscles commonly used in the field. We developed approaches allowing for experimental assessment of force production, in vitro and in vivo microscopy, and mitochondrial respiration to demonstrate the versatility of the FDB. As proof-of principle, we performed experiments to alter force production or mitochondrial respiration to validate the flexibility the FDB affords the investigator.

**Results:**

The FDB is made up of small predominantly type IIa and IIx fibers that collectively produce less peak isometric force than the extensor digitorum longus (EDL) or soleus muscles, but demonstrates a greater fatigue resistance than the EDL. Unlike the other muscles, inherent properties of the FDB muscle make it amenable to multiple in vitro- and in vivo-based microscopy methods. Due to its anatomical location, the FDB can be used in cardiotoxin-induced muscle injury protocols and is amenable to electroporation of cDNA with a high degree of efficiency allowing for an effective means of genetic manipulation. Using a novel approach, we also demonstrate methods for assessing mitochondrial respiration in the FDB, which are comparable to the commonly used gastrocnemius muscle. As proof of principle, short-term overexpression of Pgc1α in the FDB increased mitochondrial respiration rates.

**Conclusion:**

The results highlight the experimental flexibility afforded the investigator by using the FDB muscle to assess mechanisms that regulate skeletal muscle function.

## Background

Skeletal muscle is susceptible to a number of genetic, environmental, and age-related pathologies that impair the tissue’s normal mechanical and metabolic function. This often leads to the development of comorbidities and sometimes death. Defining the mechanisms that regulate the development of skeletal muscle dysfunction is critical for designing therapeutic interventions. Investigators currently employ a variety of established methods for answering such questions, but are often experimentally hampered by unique inherent heterogeneity between muscle groups and cells within the same muscle tissue. Muscles commonly used for functional and mechanistic experiments include the extensor digitorum longus (EDL), soleus, plantaris, gastrocnemius, tibialis anterior (TA), and/or the quadriceps. These muscles each offer unique advantages across a host of methodologies including measuring isometric force production, susceptibility to muscle injury, mitochondrial respiration, protein content, and histology. For example, the EDL is frequently used for measures of isometric force production or susceptibility to stretch-induced injury in order to better understand and assess the efficacy of interventions targeting Duchenne muscular dystrophy [1–3] and/or aging [4–6]. Meanwhile, studies investigating metabolic diseases such as obesity and type II diabetes commonly measure skeletal muscle mitochondrial respiration using the red portion of the gastrocnemius muscle [7–9]. However, due to inherent differences across muscles, investigators are often forced to apply specific experimental approaches for each muscle. This specificity can limit the broad interpretation of the results.

Mechanistically driven research often utilizes DNA manipulation to alter protein expression in skeletal muscle. The electroporation of cDNA or shRNA into muscles often delivers inconsistent results due to both the size of the muscle and anatomical location, which each impede uniform distribution of cDNA. These factors can lead to low transduction efficiencies, making physiological and biochemical assays unreliable. To overcome this technical limitation, investigators have tagged their gene of interest and used immunohistochemical procedures to determine changes in tagged fibers that are known to express their gene of interest. Unfortunately, this approach limits the assays that can be employed and rules out most functional assays. While transgenic models have proven a valuable resource, they remain a costly, time-consuming, and uncertain endeavor. In contrast, the flexor digitorum brevis's (FDB) unique anatomical location coupled with its size makes the muscle amenable to cDNA electroporation allowing for high transduction efficiencies.

The FDB is a skeletal muscle located in the base of the foot that has previously been used to isolate and culture single muscle fibers. For reasons that are unclear, investigators have generally limited use of the FDB to isolated fiber imaging-based approaches or satellite cell function in culture [10–13]. In this study, we demonstrate the utility of the FDB as a model for assessing skeletal muscle function across a range of methodologies commonly used within the skeletal muscle research community, including isometric force production, in vitro and in vivo imaging, and mitochondrial respiration. The full utility of the FDB is best demonstrated in studies combining these physiological tests with genetic manipulation (cDNA electroporation), thus allowing the investigator to manipulate cellular protein levels efficiently in a setting that allows for accurate functional outcome analyses.

## Methods

### Animals

All mice were 3–11-month-old C57BL/6 males at the time of testing. Mice were purchased from Jackson laboratories, housed in a temperature (22 °C) and light-controlled facility, and given free access to food and water. Mice were euthanized via isoflurane overdose. All animal procedures and usage were approved by the Institutional Review Committee at East Carolina University. Animal care complied with the Guide for the Care and Use of Laboratory Animals, Institute of Laboratory Animal Resources, Commission on Life Sciences, National Research Council (Washington: National Academy Press, 1996).

### FDB dissection

**Fig. 1:**
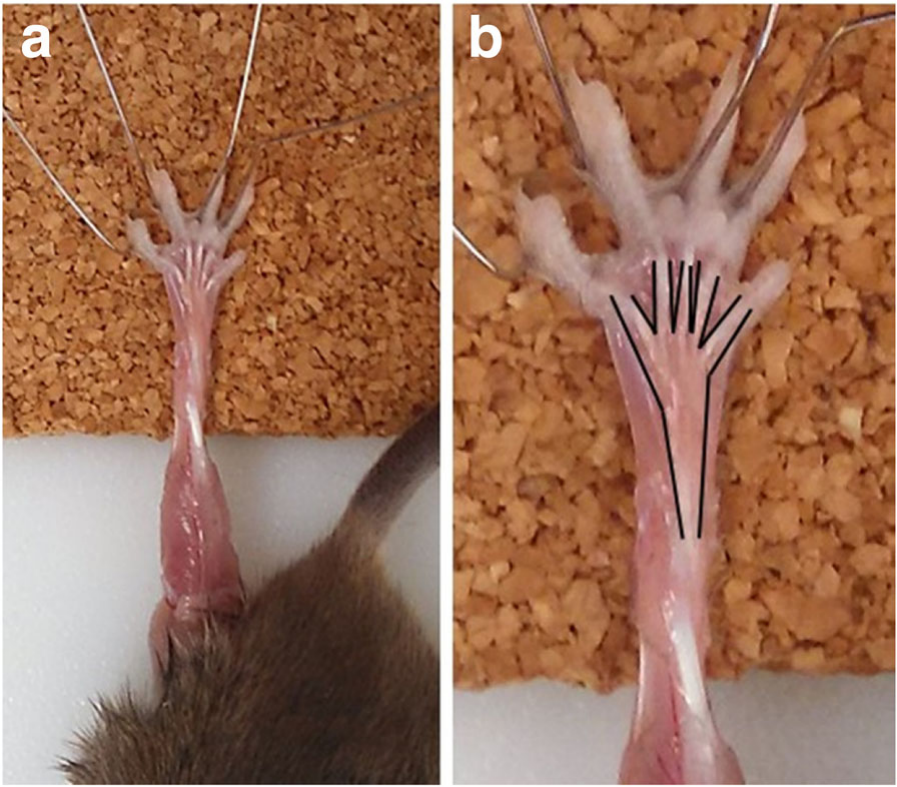
FDB dissection. **A** FDB prepared for dissection, displaying the proximal tendon and tendons of the toes. **B** Increased magnification of **A** with muscle outlined in black to define borders of muscle

Critical to any procedure utilizing the FDB is careful dissection that prevents damage to the muscle (the muscle is surrounded by an area of dense connective tissue) (see Fig. 1). To facilitate dissection, the toes were pinned to a corkboard, securing the foot in place with the sole of the foot facing upward. Next, the skin at the proximal end of the foot (above the calcaneus) was pinched allowing micro-scissors to make incisions along the lateral edges of the foot down to the toes. Still pinching the skin above the calcaneus, micro-scissors were used to separate the skin from the underlying musculature. The remaining skin flap was peeled back and removed to expose the FDB and tendons of the toes. The proximal tendon was then cut, and while holding the tendon with forceps, the FDB was cut away from the underlying fascia. The toe tendons were then cut to free the FDB from the foot.

### Myofiber isolation

After dissection of the FDB, the muscles were placed in fresh culture media (DMEM with glutamine, 2% sterile-filtered FBS, 0.1% gentamycin) supplemented with 4 mg/ml collagenase A (Roche – 11088793001) for 90–120 min at 37 °C in 5% CO_2_ as previously described [14]. The FDB muscle was placed in 2 mL of culture media without collagenase and gently triturated against the wall of the dish to release the fibers from the bundle using the cut end of a P1000 pipette tip. Isolated myofibers were adhered to glass bottom dishes that were coated with entactin-collagen-laminin (ECL Cell attachment matrix, #08110 Millipore). Fibers were returned to 37 °C in 5% CO_2_ for several hours and subsequently imaged as described below.

### FDB muscle fiber length

The FDB muscles of male and female C57BL/6 mice were tied at the proximal tendon and three medial toe tendons with silk suture and fastened to a metal clip to maintain resting tension. Myofibers were isolated as described above, but were not adhered to glass bottom plates. Myofibers were imaged using a ×4 objective and an EVOS XL core microscope and accompanying software (Life Technologies, Bothell, WA). The lengths of approximately 1000 myofibers were measured in ImageJ (version 1.6.0, NIH, Bethesda, MD). When isolated, the myofibers were no longer at tension and therefore did not represent optimal length. To account for this change in myofiber length, a conversion factor was utilized using sarcomere length, as previously described by Dr. Richard Lieber [15]. When at optimal length, the assumed mouse muscle optimal sarcomere length of a myofiber is 2.5 μm across [15]. To normalize myofiber lengths to sarcomere length, we measured sarcomere length in a subset of 30 myofibers. The distance between 10 sarcomeres of each myofiber was measured, and an average sarcomere length was calculated across all 30 myofibers. The optimal sarcomere length (2.5 μm) divided by the average measured sarcomere length produced a conversion factor of 1.14, which was used to normalize all measured myofiber lengths to optimal sarcomere length.

### Individual fiber type and fiber size

Muscle fiber type analysis of the FDB was conducted on samples from C57BL/6 mice, as previously described [16]. Sections were probed with primary antibodies against myosin heavy chain type I (BA-F8), IIa (SC-71), IIb (BF-F3) (Development Studies Hybridoma Bank, Univ of Iowa), and anti-dystrophin (Rb-9024, Thermo Fisher, Waltham, MA), then imaged using an EVOS FL auto microscope and accompanying software (Life Technologies, Bothell, WA). Fiber type and fiber cross-sectional area (CSA) were assessed using ImageJ, as previously described [16].

### Isometric force production

Isometric force production and fatigue was assessed in EDL (*n* = 5), soleus (*n* = 4), and FDB (*n* = 6) muscles of C57BL/6 mice, as previously described [17] with slight modifications. The FDB was exposed and the proximal tendon was secured using silk suture. Fine-tip forceps were placed under the three medial toe tendons and pulled gently down toward the toes before the three tendons (Fig. 1) were secured with silk suture. We tied the three medial tendons because it is not possible to tie one of the toes due to anatomical location, and tying the fourth tendon does not, in our experience, result in a different absolute force (data not shown). Each tendon was then cut just above the knot, and the FDB was gently lifted away from the foot. Micro-scissors were used to remove any remaining connective tissue, releasing it from the foot. The muscle was then tied to a force transducer and suspended in oxygenated Krebs Ringer Buffer (KRB—[mM] 115 NaCl, 2.5 KCl, 1.8 CaCl_2_, 2.2 Na_2_HPO_4_, 0.85 NaH_2_PO_4_) at room temperature. The muscle length was then adjusted until the FDB produced a peak twitch force, at which point the optimal resting tension (Lo) was set and the muscle was allowed to equilibrate for 10 min. Soleus muscles were prepared by tying a double square knot at the distal soleus tendon. The tendon was cut above the knot and the posterior muscles were gently pulled away from the leg revealing the proximal soleus tendon, which was tied with a double square knot. The EDL was dissected and tied as previously described [17]. EDL and soleus muscles were equilibrated in oxygenated room temperature KRB at resting tension for 10 min. Following equilibration, muscle tension was optimized by performing maximal twitch stimulations and adjusting the muscle length until peak force was achieved. Twitch stimulations were performed 30 s apart to avoid fatiguing the muscle. Muscles were then stimulated 60 s apart at 10, 20, 40, 60, 80, 100, and 120 Hz to generate a force frequency curve. Muscles were rested for an additional 1 min before completing a 10-min stimulation protocol to determine fatigue resistance. The fatigue protocol stimulated muscles at 30 Hz every 2 s for a period of 600 s for a total of 300 contractions. Optimal muscle length was recorded and muscles were blotted to remove excess KRB before being weighed. An optimum voltage of 20 V was established prior to the experiments to ensure maximal stimulation of the FDB, EDL, and soleus (data not shown). Absolute muscle force data were converted to specific force (N/cm^2^) using previously described equations for the mathematical estimation of muscle CSA [18] and physiological cross-sectional area (PCSA) [19]. The primary difference between CSA and PCSA is the inclusion of the muscle fiber length to muscle length ratio in the PCSA equation. We used both corrected methods to provide a wider compatibility with the literature.

### Passive contractile properties

Passive contractile properties were assessed in EDL and FDB muscles from C57BL/6 male mice (*n* = 4), as previously described [20], with slight modifications. The EDL and FDB muscles dissected and tied to a force transducer as described above. Muscles were equilibrated for 10 min in oxygenated KRB at room temperature. Following equilibration, muscle tension was optimized by performing maximal twitch stimulations and adjusting the muscle length until peak force was achieved. Twitch stimulations were performed 30 s apart to avoid fatiguing the muscle. The muscle reference length was measured as the Lo before undergoing a passive stretch of 105, 110, 115, 120, 125, and 130% of Lo. Muscles were blotted to remove excess KRB and then weighed. Data were corrected to CSA and PCSA.

### Cardiotoxin (CTX) injury

Comparisons were made in C57BL/6 mice between a CTX-treated FDB (*Naja nigricollis*, #02152238, MP Biomedicals, Santa Ana, CA) and PBS-treated contralateral FDB 4 days (*n* = 4) and 10 days post-treatment (*n* = 2). Sterile 8-mm-long 31G syringes were prepared with 10 μL of 10 μM CTX or 10 μL of sterile 1× PBS, as previously described [16]. Following isoflurane-induced anesthesia, the base of the feet of four mice was cleaned with alcohol wipes. CTX was injected at the proximal portion of the foot with the needle positioned under the skin and toward the toes. PBS was injected into the contralateral foot. Mice were sacrificed at the appropriate time, and FDBs were dissected for the measurement of force production, as described above. Data were corrected to PCSA.

### Hematoxylin and eosin staining

FDB muscles subjected to 10 days CTX treatment were flash frozen in optimal cutting temperature (OCT) solution for sectioning and H&E staining, as previously described [16].

### Skeletal muscle high-resolution mitochondrial respirometry

#### Preparation of permeabilized gastrocnemius and FDB muscle fiber bundles

Respirometry was conducted on isolated permeabilized gastrocnemius and FDB muscle bundles excised from the same limb of C57BL/6 mice (*n* = 4). A portion of the red gastrocnemius was dissected and used for the preparation of permeabilized fiber bundles, as previously described [21]. Red gastrocnemius muscle was used as the comparative tissue as it is commonly used to assess murine skeletal muscle mitochondrial respiration [8, 21]. The protocol for preparing permeabilized FDB muscle fiber bundles was adapted from previously described methods on the permeabilization of red gastrocnemius muscle bundles [21] and is outlined below. The FDBs were dissected and immediately added to ice-cold buffer X ([mM]—7.23 K_2_EGTA, 2.77 CaK_2_EGTA, 20 imidazole, 20 taurine, 5.7 ATP, 14.3 phosphocreatine, 6.56 MgCl_2_·6H_2_O, 50 MES, pH 7.1, 295 mosmol/kgH_2_O). Using a dissecting microscope, connective tissue, fat, and blood vessels were removed carefully to avoid muscle loss. FDBs were cut into bundles and divided into groups of three to four bundles weighing 1.5–2.0 mg wet weight. Bundle groups were then permeabilized in buffer X containing 22.5 μg/ml saponin with continuous rotation at 4 °C for 5 min. Muscle bundles were promptly transferred to ice-cold buffer Z ([mM]—110 K-MES, 35 KCl, 1 EGTA, 5 K_2_HPO_4_, 3 MgCl_2_·6H_2_O, 5 mg/ml BSA, pH 7.4, 295 mosmol/kgH_2_O) and washed with continuous rotation at 4 °C for 15 min.

#### Mitochondrial respiration

Measurements of high-resolution O_2_ consumption were made using the OROBOROS Oxygraph-2K (Oroboros Instruments, Innsbruck, Austria) at 37 °C with a starting oxygen concentration of ~ 300–350 μM as previously described [22]. Experiments were conducted in buffer Z containing 20 mM creatine monohydrate and 25 μM blebbistatin. Mitochondrial respiration was assessed by the sequential addition of substrates at a final concentration of pyruvate 4 mM, malate 0.5 mM, glutamate 5 mM, ADP 2.5 mM, succinate 5 mM, cytochrome c 5 μM, rotenone 10 μM, antimycin A 5 μM, ascorbic acid 2 mM, and TMPD 0.5 mM (N,N,N′,N′-tetramethyl-p-phenylenediamine dihydrochloride). Integrity of the mitochondrial membrane was confirmed by excluding gastrocnemius muscle bundles and FDB muscle bundles that produced a > 10 or > 20% increase in respiration, respectively, following exogenous cytochrome c addition. A different exclusion criteria was used for the gastrocnemius and FDB muscle bundles as preliminary testing indicated a greater percent increase in respiration was common in FDB fiber bundles compared to gastrocnemius fiber bundles, following the addition of cytochrome c. Upon completion of the protocol, muscle bundles were rinsed in distilled H_2_O, freeze-dried (Labconco, Kansas City, MO), and weighed (Orion Cahn C-35, Thermo Electron, Beverly, MA). Respiration rates for intact gastrocnemius muscle bundles are commonly corrected to dry weight; however, due to differences in connective tissue content of the two muscle groups, JO_2_ values were also corrected to total protein and citrate synthase (CS) activity. CS activity was measured using kit CS0720. Chemicals and reagents were purchased from Sigma Aldrich.

### Microscopy

#### In vitro

FDB muscle fibers were isolated from C57BL/6 mice and attached to 35-mm glass bottom dishes coated with ECL, as previously discussed. Isolated myofibers were stained with mitotracker deep red (M2246, Thermo Fisher) and NucBlue (R37605, Thermo Fisher) in DMEM for 30 min. Fibers were washed three times with 2 mL KRB. Fibers were imaged using a single photon confocal laser scanning microscope with a ×60 oil immersion objective (Olympus, Plan Apochromat, NA 1.35) and excitation was achieved using the 405- and 488-nm lines of a multiline argon laser.

#### In vivo

Second harmonic generation describes the optical effect produced from the passage of laser pulses through highly polarized, non-centro-symmetrical materials such as myosin. When polarized at the appropriate wavelength, these materials emit light at half the wavelength of that entering, producing high-resolution images without the need for fluorescent probes that are subject to photobleaching and phototoxicity. Furthermore, the near infrared wavelengths used allow for deep tissue penetration without the need for invasive procedures [23]. C57BL/6 mice were anesthetized before having the skin covering the FDB removed, exposing the FDB muscle. Once exposed, the FDB was hydrated with sterile KRB and the mouse was laid prone on a glass cover slip (#1.5, Leica), as previously described [24] (Fig. 1C). Myosin and nicotinamide-containing molecules were excited at 900 and 720 nm using a mode locked Ti:Sapphire pulsed laser (Mai Tai Deep See HP series, Spectra-Physics, Santa Clasa, CA), and emission was recorded using non-descanned detection with a FV10 MRV/G filter set at 450 and 420 nm, respectively. All images were taken using a ×60 oil immersion objective (Plan Apochromat, NA 1.35) and an Olympus FV1000 LSM operating FV10-ASW 4.2 acquisition software.

#### cDNA electroporation and high-resolution respirometry of skeletal muscle overexpressing Pgc1α

Electroporation of cDNA into FDBs was performed as previously described [25]. Briefly, the feet of seven C57BL/6 male and female anesthetized mice were cleaned with an alcohol wipe and the footpads were injected with 10 μl of 2 mg/mL hyaluronidase suspended in sterile-filtered KRB using an 8-mm-long 31 gauge sterile needle. Approximately 1 h, later mice underwent anesthesia for a second time. Feet were again cleaned with an alcohol wipe and one foot received 30 μg of green fluorescent protein (GFP)-tagged *PGC1α* plasmid and the contralateral foot was injected with *YFP* cDNA. Following a full recovery from anesthesia, mice were anesthetized for a third time ~ 10 min later. Platinum electrodes were inserted under the skin and positioned perpendicular to the FDB and parallel to one another at the heel and footpad beneath the toes. The FDB was stimulated with 20 pulses of 20 ms duration at 1 Hz and 100 V [25]. Mice were sacrificed 14 days later, and FDBs were dissected and imaged under a fluorescent microscope to confirm plasmid expression. Intact FDB muscle samples were then prepared and measured for mitochondrial respiration as outline above.

#### Statistics

Data distributions were assessed and data that did not conform to a normal distribution were log base 10 transformed. Force frequency contractile data were analyzed via two-way ANOVA with Tukey multiple comparisons. Muscle mass, fiber type, time to max and half-relaxation, and fatigue data were analyzed via one-way ANOVA with Tukey multiple comparisons. In cases where data could not be transformed to a normal distribution, a Kruskal-Wallis test with Dunn multiple comparisons was performed. ANOVAs were completed using GraphPad Prism 7.03. Respiratory data and CS activity were analyzed via paired two-tailed *t*-tests with an alpha level of 0.05 using Microsoft Excel. All data are presented as mean ± SEM.

## Results

### FDB fiber type and comparative characteristics

**Fig. 2:**
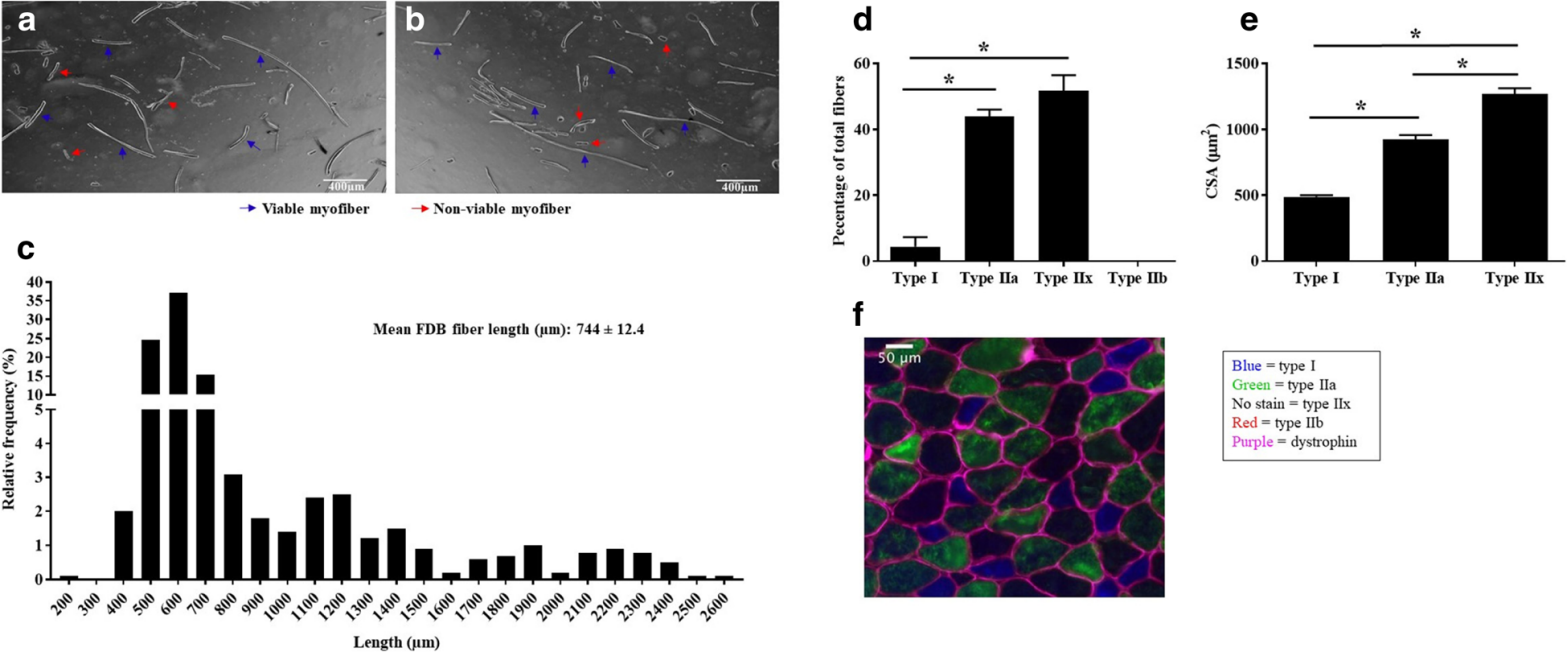
FDB fiber size, type, and cross-sectional area (CSA). **A**, **B** Example images of single myofibers isolated from FDB muscle. Blue arrows indicate viable myofibers. Red arrows indicate non-viable myofibers. Only viable myofiber lengths were measured. **C** Relative frequency of FDB muscle fiber lengths. **D** Fiber type percentage of whole FDB muscles (*n* = 4). **E** Average individual CSA (μm^2^) of each muscle fiber type in the FDB. **F** Representative FDB cross section stained for type I (blue), type IIa (green), type IIx (no stain), type IIb (red), and dystrophin (purple). *, *p* < 0.05. Data are mean ± SEM

Phenotypic characterization of the FDB demonstrates it is a smaller muscle than the EDL and soleus and displays a smaller fiber length to muscle length ratio relative to the EDL and soleus, as measured by Brooks and Faulkner [19] (Table 1). The FDB myofiber length is highly variable (Fig. 2A, B), although an analysis of the myofiber length frequency indicates a majority of the myofibers are approximately 500–700 μm (Fig. 2C). The FDB exhibits a mixed muscle fiber type comprising of predominantly type IIa fibers (43.9 ± 1.9%) and type IIx fibers (51.6 ± 4.8%) with a small population of type I fibers (4.4 ± 2.9%) (Fig. 2D). The cross-sectional area (CSA) of the individual muscle fiber types were all significantly different, with the largest CSA found in type IIx fibers, followed by IIa and I, respectively (Fig. 2E, F).

**Table 1:** Characteristics of the FDB compared to the EDL and soleus muscles

	FDB	EDL	Soleus
Muscle mass (mg)	6.7 ± 0.4*	11.2 ± 0.7	10.0 ± 0.4
Optimal muscle length (mm)	9.5 ± 0.2*	14.2 ± 0.3	13.4 ± 0.3
Fiber length (mm)	0.744 ± 0.01	5.44 ± 0.12 [19]	7.84 ± 0.22 [19]
Fiber to muscle length ratio	0.079 ± 0.002	0.45 ± 0.004 [19]	0.69 ± 0.006 [19]
Fiber type %			
- Type I	4.4 ± 2.9	0	35
- Type IIa	43.9 ± 1.9	3	53
- Type IIx	51.6 ± 4.8	25	11
- Type IIb	0	72	1
		[26]	[26]
CSA (μm^2^ ± SD)			
- Type I	488 ± 67	–	943 ± 198
- Type IIa	927 ± 314	634 ± 142‡	790 ± 149‡
- Type IIx	1267 ± 462	–	–
- Type IIb	–	772 ± 120	–
		[35]	[35]

Numbers in [brackets] represent the data citation

*FDB v.s. EDL; *p* < 0.05

‡Figure represents a combination of type IIa and type IIx muscle fibers. Data are mean ± SEM unless otherwise stated

### Isometric contractions and force production

**Fig. 3:**
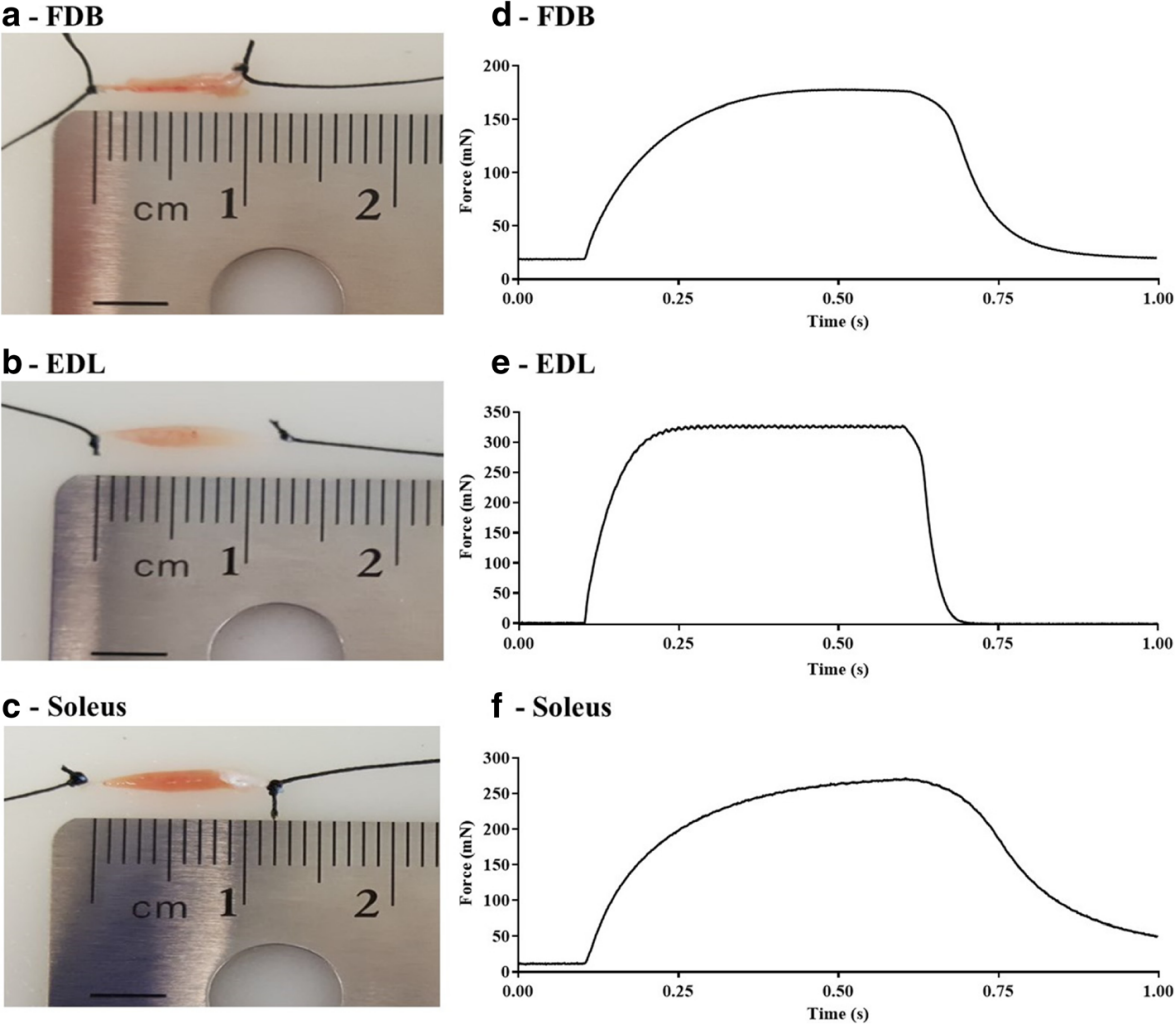
Comparison of FDB, EDL, and soleus muscle length, and in vitro isometric contraction profile. Example images of **A** FDB, **B** EDL, and **C** soleus muscle after the tendons were secured with silk suture for subsequent isometric force assessment. Example tetanic (100 Hz) tracings for each muscle: **D** FDB, **E** EDL, **F** soleus

**Fig. 4:**
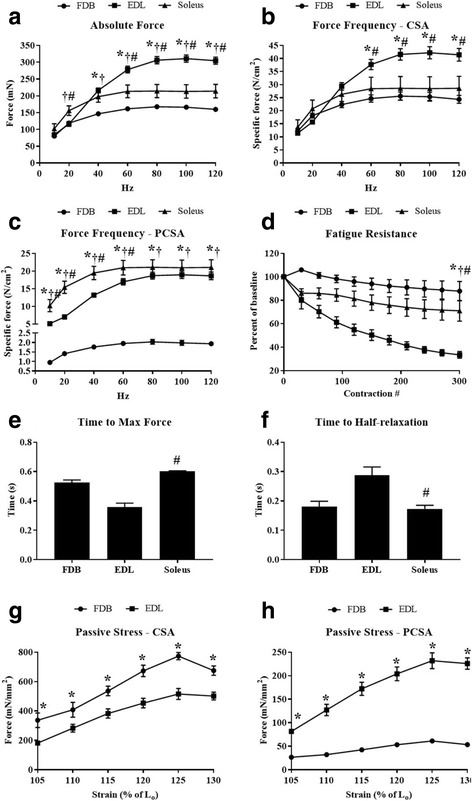
Comparison of in vitro force production characteristics and fatigue resistance. Force frequency curves were generated from FDB (*n* = 6), EDL (*n* = 5), and soleus muscles (*n* = 4). **A** Absolute force (mN). **B** Specific force (N/cm^2^) corrected to CSA. **C** Specific force (N/cm^2^) corrected to PCSA. **D** Percent decline in baseline force during a 10-min fatigue resistance protocol. **E** Time (s) to maximum force. **F** Half-relaxation time (s). Time to maximum force and half-relaxation times for each muscle were averaged from 100 Hz contractions. **F–H** Comparison of passive stress lengthening contractions between the FDB and EDL (mN/mm^2^) corrected to CSA and PCSA, respectively. *, FDB v.s. EDL *p* < 0.05; †, FDB v.s. soleus *p* < 0.05; #, EDL v.s. soleus *p* < 0.05. Data are mean ± SEM

The respective whole muscle length and contractile kinetics of the FDB, EDL, and soleus are demonstrated in Fig. 3A–F. The FDB produces less peak force than either the EDL or soleus muscle (Fig. 4A–C). The comparative relationship between the FDB, EDL, and soleus specific force is highly dependent on whether force is normalized to CSA (Fig. 4B) or PCSA (Fig. 4C). The FDB is more fatigue resistant than the EDL and the soleus (Fig. 4D). The FDB exhibits differing contractile kinetics compared to the EDL. Relative to the soleus, the FDB displays a statistically similar time to max force and half-relaxation time (Fig. 4E, F).

### Passive contractile properties

Using a previously described protocol [20] with slight modifications, we assessed the passive mechanical properties of the FDB and EDL muscles. In response to a graded increase in muscle length (strain), both the FDB and EDL recorded increasing force (mN/mm^2^) at each length up to 125% of Lo. At 130% Lo, both the FDB and EDL did not demonstrate any further increases in force output compared to 125% Lo. The relationship between the FDB and EDL in response to muscle lengthening was substantially altered by the method of force normalization. When normalized to CSA, the FDB consistently exhibited significantly more force than the EDL (Fig. 4G). However, when normalized to PCSA (accounting for the respective fiber length to muscle length ratios), the EDL exhibited significantly more force than the FDB (Fig. 4H).

### CTX-induced muscle injury prevents force production

**Fig. 5:**
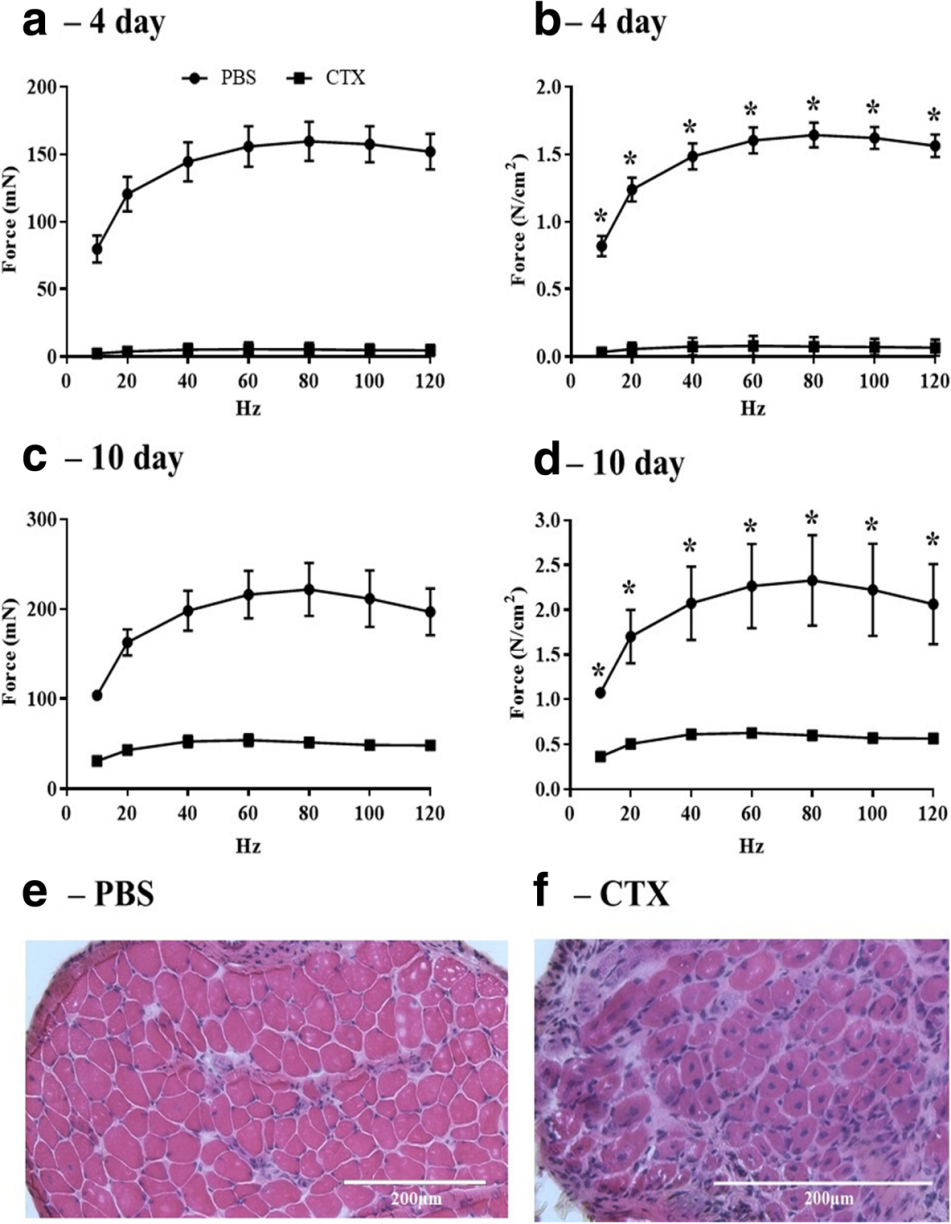
CTX-induced muscle injury. Mice were injected with 10 μL of 10 μM CTX in one FDB and 10 μL sterile PBS in the contralateral FDB. Mice were euthanized 4 days (*n* = 4) or 10 days (*n* = 2) post-injection. FDBs were dissected and force production was recorded for control and injured muscles using a force-frequency curve protocol. FDBs tested 10 days post-treatment were flash frozen in OCT, sectioned to 12 μm, and stained with H&E before imaging at ×20. **A** Four days post-injection absolute force (mN). **B** Four days post-injection specific force (N/cm^2^) corrected to PCSA. **C** Ten days post-injection absolute force (mN). **D** Ten days post-injection specific force (N/cm^2^) corrected to PCSA. **E** PBS-treated FDB muscle section stained with H&E. **F** CTX-treated FDB muscle section stained with H&E. *, *p* < 0.05. Data are mean ± SEM

FDB muscles injected with CTX failed to produce measurable force at 4 days post injection (Fig. 5A, B). However, force began to recover by 10 days post injection, although force production still remained impaired relative to contralateral control limbs (Fig. 5C, D). H&E-stained muscle sections showed a greater number of nuclei, which were predominantly centrally located, in FDB muscle injected with CTX, compared to PBS-injected FDB muscle sections, following 10 days of recovery (Fig. 5E, F).

### Comparison of mitochondrial respiration between red gastrocnemius and FDB muscle

**Fig. 6:**
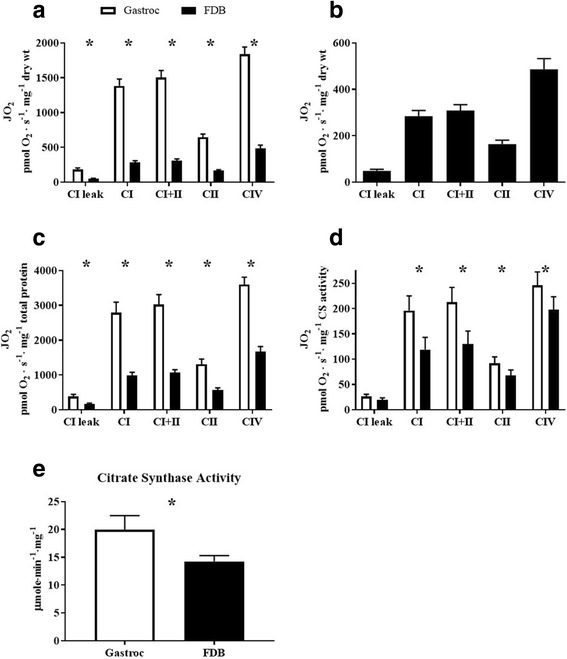
Comparison of mitochondrial respiration between the FDB and red gastrocnemius muscle. Permeabilized FDB and gastrocnemius muscle bundles were prepared for high-resolution respirometry (*n* = 4). Substrates and inhibitors were added sequentially to measure CI leak and ADP-stimulated CI, CI + II, CII, and CIV mitochondrial respiration. **A** Comparison of mitochondrial respiration in intact permeabilized red gastrocnemius and FDB muscle bundles corrected to dry weight. **B** Inset of graph **A** showing FDB mitochondrial respiration only. **C** Comparison of mitochondrial respiration in intact permeabilized red gastrocnemius and FDB muscle bundles corrected to total protein content. **D** Comparison of mitochondrial respiration in intact permeabilized red gastrocnemius and FDB muscle bundles corrected to CS activity. **E** Comparison of CS activity between red gastrocnemius and FDB muscle. CI leak, complex I leak^state4^; CI, complex I respiration^state3ADP^; CI + II, complex I and II respiration^state3ADP^; CII, complex II respiration^state3ADP^; CIV, super-complex respiration. *, *p* < 0.05. Data are mean ± SEM

Mitochondrial respiration rates for complex I leak^state 4^, complex I^state3ADP^, complex I and II^state3ADP^, complex II^state3ADP^, and complex IV were consistently higher in red gastrocnemius muscle compared to FDB muscle (Fig. 6A). FDB mitochondrial respiratory data, normalized to muscle dry weight, is displayed alone in Fig. 6B. The magnitude of the difference in respiration between red gastrocnemius and FDB muscle was dependent on the corrective method employed. Correcting to muscle dry weight demonstrated the greatest relative difference in respiration between the two muscles (Fig. 6A). Meanwhile, correcting mitochondrial respiration to total cellular protein reduced the relative difference in respiration (Fig. 6C), which was reduced further by correcting mitochondrial respiration to CS activity (Fig. 6D). Gastrocnemius mitochondrial respiration was significantly greater across all complexes and conditions when corrected to muscle dry weight and total protein, while complex I leak^state 4^ was no longer significant when corrected to CS activity. CS activity was greater (*p* = 0.04) in red gastrocnemius muscle compared to FDB muscle (Fig. 6E), suggesting that the red gastrocnemius muscle has a higher mitochondrial content then the FDB muscle.

### In vitro and in vivo microscopy

**Fig. 7:**
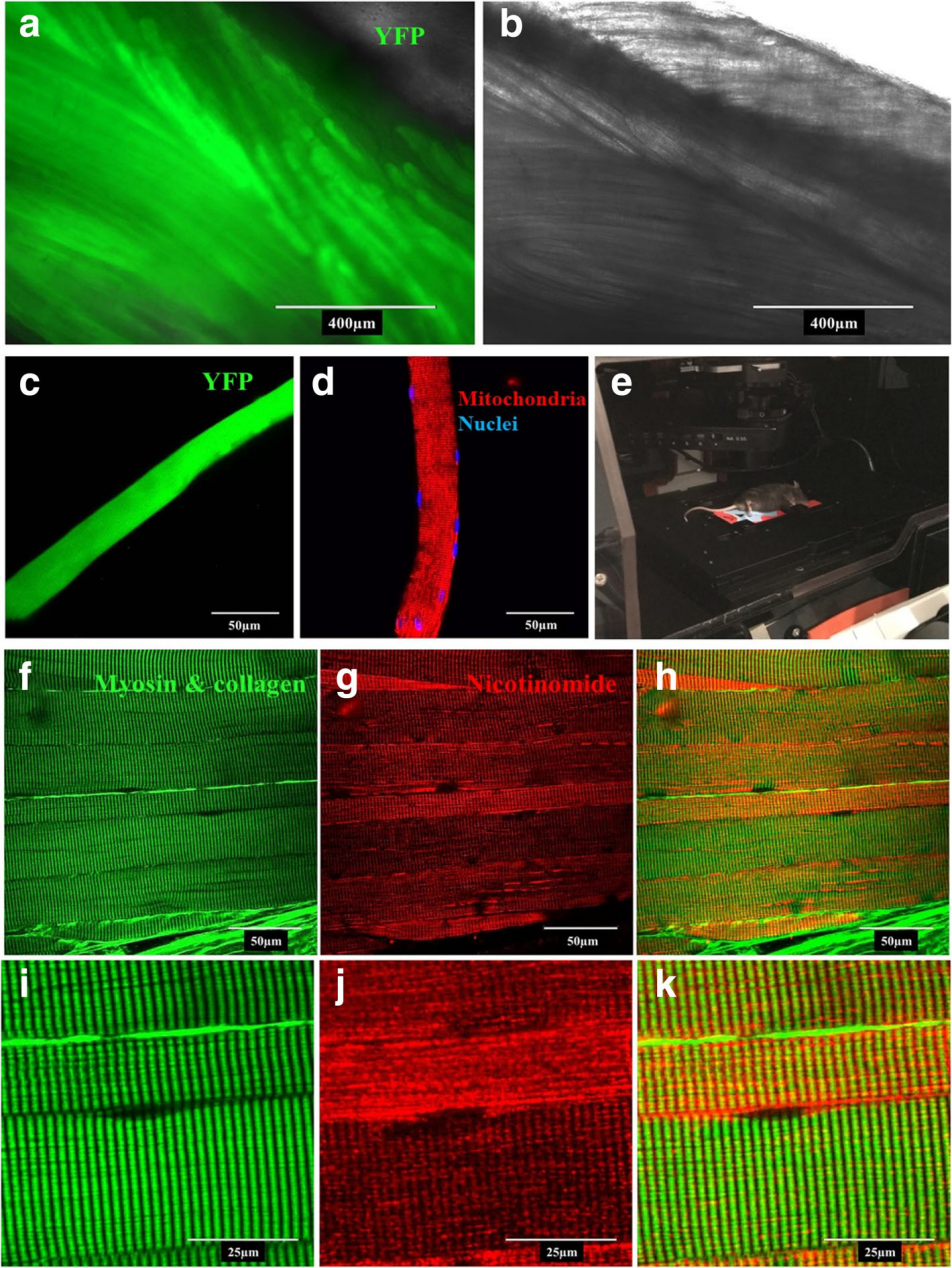
In vitro and In vivo imagining. In vitro imaging techniques were used to demonstrate the expression of YFP protein in whole FDB muscle 7 days post cDNA electroporation, and the retention of YFP post-myofiber isolation. **A** Whole FDB muscle expressing YFP; images were taken with a 488-nm filter cube and in phase contrast then merged to show transfection efficiency. **B** Whole FDB muscle YFP negative control; images were taken with a 488-nm filter cube and in phase contrast then merged. **C** Isolated FDB myofiber demonstrating retention of YFP. FDB myofibers were isolated and stained with mitotracker deep red and DAPI prior to imaging with a single photon confocal LSM and oil immersion ×60 objective. **D** Representative images of stained isolated FDB myofibers showing mitochondria in red and nuclei in blue. Two-photon excitation fluorescence microscopy and second generation harmonics were employed to generate high-resolution in vivo images of the FDB myosin structure (green) and nicotinamide-containing molecules (red) using an oil immersion ×60 objective. Representative images displayed. **E** Photograph of mouse being prepared for second harmonic generation microscopy of the FDB. The mouse has been tilted slightly to better show the fixing of the foot to the slide. Images were captured with the mouse in a prone position. **F** Myosin. **G** Nicotinamide. **H** Composite of **F** and **G**. **I** ×3 zoom inset of **F**. **J** ×3 zoom inset of **G**. **K** Composite of **I** and **J**

Here we demonstrate the FDB muscle is amenable to multiple imaging approaches. First, we took advantage of the susceptibility of the FDB to cDNA electroporation to demonstrate fluorescent imaging approaches using the whole FDB muscle (Fig. 7A, B) and single muscle fibers isolated from the FDB (Fig. 7C). It is possible to determine localization of tagged proteins with in vitro imaging options using commercially available dyes such as DAPI and mitotracker deep red (MTDR) (Fig. 7D). However, it is also possible to demonstrate cellular localization when imaging the whole FDB muscle by using second harmonic generation microscopy. Figure 7E shows a mouse being prepared for second harmonic generation microscopy. Our results demonstrate high-resolution label-free images where we were able to image myosin/collagen and/or NAD(P)H at the single cell level while the muscle remained intact (Fig. 7F–K).

### cDNA electroporation-induced elevation of Pgc1α increases mitochondrial respiration in intact FDB muscle

**Fig. 8:**
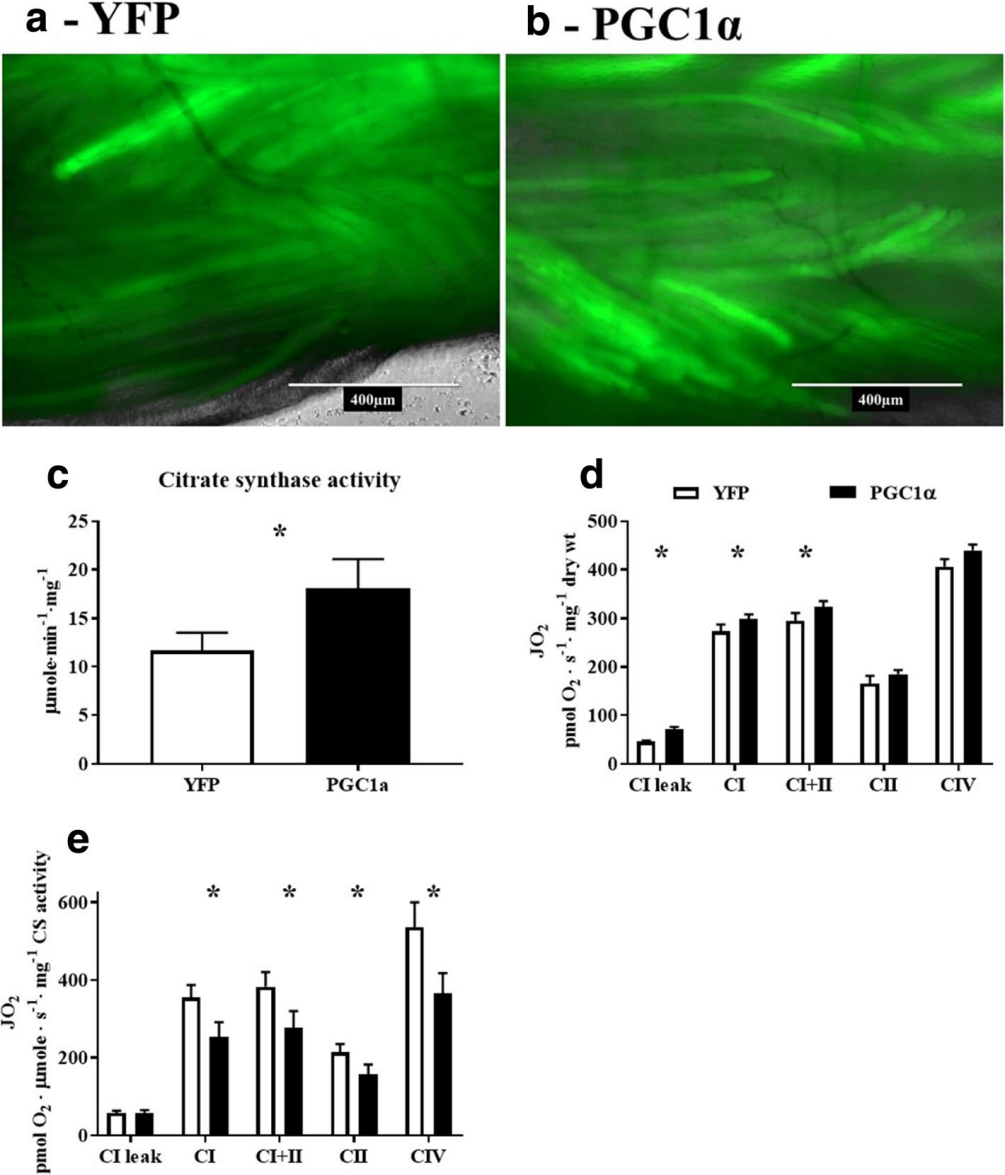
Electroporation of *PGC1α* cDNA increased mitochondrial respiration. GFP-conjugated *PGC1α* and *YFP* cDNA were electroporated into one FDB and a contralateral control FDB, respectively (*n* = 7). Mice were euthanized 14 days later and whole FDB muscle was imaged using fluorescent microscopy to confirm protein expression before intact permeabilized FDB muscle bundles were prepared for assessment of mitochondrial respiration between FDBs overexpressing Pgc1α or YFP. **A** YFP expression in whole FDB 14 days post cDNA electroporation; images were taken with a 488-nm filter cube and in phase contrast then merged to show transfection efficiency. **B** GFP-conjugated Pgc1α expression in whole FDB 14 days post cDNA electroporation; images were taken with a 488-nm filter cube and in phase contrast then merged to show transfection efficiency. **C** Comparison of CS activity between YFP and Pgc1α overexpressing FDB muscle bundles. **D** Overview of mitochondrial respiration across ETC protein complexes between YFP and Pgc1α overexpressing FDB muscle bundles, corrected to muscle dry weight. **E** Overview of mitochondrial respiration across ETC protein complexes between YFP and Pgc1α overexpressing FDB muscle bundles, corrected to CS activity. CI leak, complex I leak^state 4^; CI, complex I respiration^state3ADP^; CI + II, complex I and II respiration^state3ADP^; CII, complex II respiration^state3ADP^; CIV, super-complex respiration. * *p* < 0.05. Data are mean ± SEM

Fluorescent microscopy of whole FDB muscle confirmed the effective expression of both GFP-tagged Pgc1α in one foot and YFP in the contralateral foot (Fig. 8A, B). Additionally, we confirmed the successful upregulation of mitochondrial biogenesis by measuring CS activity. FDB muscle overexpressing Pgc1α demonstrated significantly greater CS activity when compared to FDB muscle expressing YFP (Fig. 8C). Overexpression of Pgc1α significantly increased mitochondrial respiration in the FDB muscle compared to the contralateral FDB electroporated with YFP (Fig. 8D). Specifically, complex I leak respiration (*p* = 0.002), ADP-stimulated complex I (*p* = 0.049), and complex I + II (*p* = 0.038) respiration was higher in Pgc1α overexpressing FDB muscle compared to YFP-expressing FDBs. ADP-stimulated complex II (*p* = 0.080) respiration as well as complex IV (*p* = 0.069) respiration were not significantly elevated. Interestingly, normalizing oxygen consumption to CS activity largely abolished the differences between YFP and Pgc1α-treated FDBs, thereby confirming that increased respiration observed in Pgc1α-treated FDBs was due solely to increased mitochondrial content (Fig. 8E).

## Discussion

In these experiments, we demonstrate the broad versatility of the FDB muscle as a potential tool for the assessment and investigation of skeletal muscle biology. Our findings validate the FDB muscle across a range of methodologies and highlight its inherent experimental advantages. The location of the FDB, beneath the skin, and relatively small size provide a means to accurately target and efficiently transfect cDNA for the manipulation of protein expression. Importantly, the FDB can then be used across a variety of physiological and/or biochemical-based assays. As proof of principle, in the experiments described here, we specifically demonstrate that electroporation of cDNA encoding *PGC1α* significantly increased mitochondrial respiration.

To provide investigators a better understanding of the FDB, we performed a comprehensive phenotypic description compared to the EDL and/or soleus muscle. A novel aspect of the current study is that to the knowledge of the authors’ this is the first study to measure the muscle fiber length to muscle length ratio in the FDB of mice. This provides an important advancement in the ability to accurately compare the force-producing capacity of the FDB with other muscles, such as the EDL and soleus, for which fiber length to muscle length ratios were previously known [19]. In the past, and in the absence of a fiber length ratio for murine FDB muscle, force has been normalized without accounting for muscle fiber lengths [18]. This method of normalization has been employed in other investigations across other muscles such as the soleus and EDL. We have therefore normalized our data using both approaches. However, it may be important to account for the fiber length to muscle length ratio as any intervention that could affect either variable would alter force output. Examination of the force frequency curves between FDB, EDL, and soleus muscles, or the FDB and EDL muscles following passive stress exemplifies the substantial effect of accounting for the muscle fiber to muscle length ratio. Researchers should be mindful of the normalization method used when analyzing and interpreting data, and when comparing to data in the literature.

We observed an intermediate contractile phenotype in the FDB that quantitatively places it between the EDL and soleus muscles with respect to contractile kinetics. Not surprisingly, the contractile kinetics of the FDB mirror the fiber type composition, which consists predominantly of types IIx and IIa with a small percentage of type I fibers. When compared to the soleus muscle (type *I* = 35%, IIa = 53% and IIx = 11%, IIb = 1%) or the EDL (type *I* = 0%, type IIa = 3%, IIx = 25% and IIb = 72%) [26], the FDB presents as a more mixed phenotype. It is worth noting that the greater fatigue resistance of the FDB muscle may not be the direct result of biochemical differences, but an indirect result of a reduced oxygen diffusion distance, resulting from the smaller size of the FDB relative to the soleus and EDL. In conjunction with the EDL and soleus, the FDB provides an additional muscle in which functional outcomes can be assessed in response to experimental manipulations. Due to the unique anatomical location, the FDB is amenable to direct injections of various small molecules and/or toxins. As proof of principle, we show that the loss and recovery of isometric force production within the FDB following CTX-induced muscle injury is similar to that previously reported in the EDL [27]. Together, these data suggest the FDB may be a valuable addition to, or substitution for, the EDL and soleus muscles when assessing skeletal muscle force production and contractile kinetics.

The FDB has a smaller fiber: muscle length ratio relative to the EDL. This ratio makes the FDB amenable to efficient single myofiber isolation, which is more challenging in the EDL and/or soleus muscles. These single myofibers can be cultured and imaged on multiple different microscopy platforms. Skeletal muscle microscopy is a valuable tool for identifying protein localization [28], muscle morphology [29], and the dynamic changes in molecules such as H_2_O_2_ [10, 30] and calcium [29, 31]. In vitro imaging of isolated FDB myofibers is well-established; however, our findings demonstrate that electroporated cDNA tagged with fluorescent proteins is retained in myofibers following isolation. This provides numerous possibilities for the manipulation of protein expression and examination of subsequent alterations to morphology and function. In addition, the combination of fluorescently labeled cDNA and specific dyes or fluorophores, such as MTDR and DAPI, may be used to image protein localization. These techniques may be further enhanced through in vivo imaging. We specifically demonstrate how two-photon confocal microscopy and principles of second harmonic generation allow for the high-resolution imaging of non-centro-symmetrical materials such as muscle myosin, nicotinamide-containing molecules, and collagen [23]. The FDB muscle provides several advantages for using second harmonic generation microscopy. The natural prone posture of the animal may aid in reducing motion artifact due to breathing. Imaging the FDB in the fashion described is less invasive than alternative methods, such as excision of the cremaster [32]. The amenable nature of the FDB to electroporation with fluorescently tagged cDNA may also allow for a combination of single- and two-photon microscopy, generating high-definition protein localization images.

In recent years, there has been a substantial increase in the utilization of methods assessing mitochondrial respiratory function, particularly so within the field of skeletal muscle-related metabolic disease. We demonstrate a novel approach, utilizing high-resolution respirometry to measure mitochondrial respiration in the FDB muscle. To validate the use of the FDB, we compared mitochondrial respiration rates between red gastrocnemius muscle and the FDB. As anticipated, respiration rates were higher in red gastrocnemius muscle. This is in part due to differences in mitochondrial content as demonstrated by greater CS activity, but also a result of the respective tissue preparation protocols. The magnitude of the difference in respiration rates was dependent on whether O_2_ consumption was normalized to muscle dry weight, total protein, or CS activity. Normalizing to dry weight or total protein altered respiration values as the FDB contains a relatively higher content of connective tissue compared to the gastrocnemius, thus leading to potential underestimation of FDB O_2_ consumption rates. While adjusting the method of normalization cannot fully account for differences in connective tissue content, when rates were normalized to CS activity, this possible underestimation was eliminated. This suggests that strong consideration to normalization must be given prior to starting any study with the FDB. CS activity was selected as an alternative normalization factor for O_2_ consumption rates due to its strong association with mitochondrial content [33]. Further normalization methods, such as mitochondrial protein complex content, may be used, but interactions between the normalization factor and treatment should be considered. Investigators should also consider the size of the FDB in their study designs as this represents a limiting factor to the number of protocols and replicates that may be tested. Additionally, the FDB is too sensitive to standard mechanical separation of muscle fibers, so this should be avoided to protect mitochondrial membrane integrity.

Finally, as an overall proof of concept that the FDB affords the investigator a unique flexibility, we overexpressed GFP-tagged Pgc1α or YFP protein in the FDB for a period of 14 days using cDNA electroporation. A fluorescence imaging-based approach in combination with a CS activity assay confirmed the successful overexpressing of Pgc1α. The significantly greater complex I^LEAK^, complex I^ADP^, and complex I + II^ADP^ respiration rates in GFP-tagged Pgc1α-injected FDBs demonstrates that the electroporation of cDNA into FDBs is an effective means of manipulating the phenotype or function of the muscle. Normalizing oxygen consumption rates to CS activity resulted in no group differences compared to dry muscle weight normalization. These data suggest that Pgc1α may have a larger influence on CS activity relative to the protein complexes of the electron transport chain (ETC). More specifically, each complex of the ETC is made up of numerous proteins that are likely not all regulated by Pgc1α suggesting the necessity of additional regulators of mitochondrial function [34].

While there are practical advantages to using the EDL, soleus, or gastrocnemius muscles for assessing skeletal muscle isometric force production or mitochondrial respiration, these muscles are not effective targets for cDNA electroporation due primarily to their size and/or anatomical location (which impedes cDNA distribution within the muscles). Conducting mechanistic studies into the relationships between protein expression and function using such muscles therefore requires the production of muscle-specific transgenic mice. The FDB provides a time sensitive, non-invasive, and cost-effective method for assessing multiple functional indices within skeletal muscle after deliberately altering protein expression through cDNA or shRNA delivery. This combined utility provides advantages to investigators pursuing mechanistic studies in the field of skeletal muscle biology.

## Conclusions

The use of the FDB represents an expanded tool set to the mechanism-focused investigator. We demonstrate that the FDB may act as a substitute for a number of commonly assessed skeletal muscles across a range of methodologies. In addition, the versatility of the FDB supports a number of complementary methodologies that may advance the assessment of skeletal muscle function and aid in elucidating the underlying mechanisms central to understanding skeletal muscle-related genetic disorders and disease.

## Abbreviations

cDNA: Complementary DNA
CS: Citrate synthase
CSA: Cross-sectional area
CTX: Cardiotoxin
EDL: Extensor digitorum longus
ETC: Electron transport chain
FDB: Flexor digitorum brevis
GFP: Green fluorescent protein
KRB: Krebs Ringer Buffer
MTDR: Mitotracker deep red
PCSA: Physiological cross-sectional area
Pgc1α: Peroxisome proliferator-activated receptor gamma coactivator 1-alpha
YFP: Yellow fluorescent protein
